# Supplementary material for: Sorting at embryonic boundaries requires high heterotypic interfacial tension
Source: Nat Commun. 2017 Jul 31;8:157. doi: 10.1038/s41467-017-00146-x (PMC5537356; doi:10.1038/s41467-017-00146-x)
Supplement: Supplementary file 2 — Supplementary Software 1 [file 41467_2017_146_MOESM2_ESM.zip › PottsModel/SrcPottsModel/doc/index-files/index-9.html]

I-Index


JavaScript is disabled on your browser.


Skip navigation links


- Overview
- Package
- Class
- Use
- Tree
- Deprecated
- Index
- Help

- Prev Letter
- Next Letter

- Frames
- No Frames

- All Classes

A B C D E F G H I J K L M N O P R S T U V W 


## I

IEnergyFunction - Interface in model


increaseFactorAdhesion - Variable in class model.Constants.ParameterManager


increaseFactorTemperature - Variable in class model.Constants.ParameterManager


increaseTime - Variable in class model.Constants.ParameterManager


initalize() - Method in class model.Cell


initialize() - Method in class model.Pixel


initializeMatrix(Lattice) - Method in class gui.PottsCanvas


initializeMatrix(Lattice) - Method in class gui.SnapshotManager


InteractionEnergyStatistic - Class in engine


InteractionEnergyStatistic(PottsEngine) - Constructor for class engine.InteractionEnergyStatistic


InteractionEnergyStatistic(PottsEngine, int) - Constructor for class engine.InteractionEnergyStatistic


InterfacialTensionEnergyFunction - Class in model


InterfacialTensionEnergyFunction() - Constructor for class model.InterfacialTensionEnergyFunction


inverse(Constants) - Static method in class model.Lattice


inverse(int[][][]) - Static method in class model.LatticeUtilities


inverse(Cell.CellType) - Static method in class model.LatticeUtilities


IO - Class in utils


IO() - Constructor for class utils.IO


IObservable - Interface in mvc


IPositionManager - Interface in model


IPositionManager.Position - Enum in model


isActive(PixelShape.Edge) - Method in class gui.PixelDisplay


isAppendToCSV() - Method in class model.Constants


isAreaRendered() - Method in class engine.Statistic


isAreaStatisticTracked() - Method in class model.Constants


isAutomaticMode() - Method in class model.Constants


isBoundary() - Method in class model.Pixel


isCellCoordinatesCSVStatisticTracked() - Method in class model.Constants


isCellEnergyStatisticTracked() - Method in class model.Constants


isCellShapeCSVStatisticTracked() - Method in class model.Constants


isCsvSaved() - Method in class model.Constants


isDebugMode() - Method in class model.Constants


isDeltaEnergyTracked() - Method in class engine.StatisticsManager


isDispersionIndexStatisticTracked() - Method in class model.Constants


isDisplayed() - Method in class engine.CSVStatistic


isDisplayed() - Method in class engine.Statistic


isEnabled() - Method in class model.Constants


isGraphicalOutputEnabled() - Method in class model.Constants


isHblStatisticTracked() - Method in class model.Constants


isHMDStatisticTracked() - Method in class model.Constants


isInSameCell(Pixel) - Method in class model.Pixel


isIsolated() - Method in class model.Cell


isIsolated(CellSet) - Method in class model.Cell


isIsoperimetricQuotientStatisticTracked() - Method in class model.Constants


isLatticeSaved() - Method in class model.Constants


isMedium() - Method in class model.Cell


isMedium() - Method in class model.Pixel


isNeighborsTracked() - Method in class model.Constants


isNeighborWith(Pixel) - Method in class model.Pixel


isNNStatisticTracked() - Method in class model.Constants


IsoperimetricQuotientStatistic - Class in engine


IsoperimetricQuotientStatistic(PottsEngine, Cell.CellType[], int) - Constructor for class engine.IsoperimetricQuotientStatistic


isPerimeterStatisticTracked() - Method in class model.Constants


isRandomCellTypes() - Method in class model.Constants


isSaved() - Method in class model.Constants
:   Is anything being saved?

isScalePixelSize() - Method in class model.Constants


isSnapshotsSaved() - Method in class model.Constants


isSpinAttemptsObserved() - Method in class engine.CellCoordinatesCSVStatistic


isSpinAttemptsObserved() - Method in class engine.CellShapeCSVStatistic


isSpinAttemptsObserved() - Method in class engine.CSVStatistic


isSpinAttemptsObserved() - Method in class engine.Statistic


isSpinAttemptsObserved() - Method in class engine.StatisticsManager


isStateObservable(PottsEngine.State) - Method in class engine.CellCoordinatesCSVStatistic


isStateObservable(PottsEngine.State) - Method in class engine.CellShapeCSVStatistic


isStateObservable(PottsEngine.State) - Method in class engine.CSVStatistic


isSuccessfulSpinCopyAttemptsTracked() - Method in class model.Constants


isValidEdge(PixelShape.Edge) - Method in class gui.HexagonPixelDisplay


isValidEdge(PixelShape.Edge) - Method in class gui.PixelDisplay


isValidEdge(PixelShape.Edge) - Method in class gui.SquarePixelDisplay


iterator() - Method in class model.CellSet


iterator() - Method in class model.Lattice

A B C D E F G H I J K L M N O P R S T U V W

Skip navigation links


- Overview
- Package
- Class
- Use
- Tree
- Deprecated
- Index
- Help

- Prev Letter
- Next Letter

- Frames
- No Frames

- All Classes
